# Enhancing the Quality of Polypropylene Recyclates: Predictive Modelling of the Melt Flow Rate and Shear Viscosity

**DOI:** 10.3390/polym16162326

**Published:** 2024-08-16

**Authors:** Lukas Seifert, Lisa Leuchtenberger-Engel, Christian Hopmann

**Affiliations:** Institute for Plastics Processing (IKV) in Industry and Craft at RWTH Aachen University, Seffenter Weg 201, 52074 Aachen, Germany

**Keywords:** polypropylene, recycling, Melt Flow Rate, shear viscosity, symbolic regression, predictive modelling

## Abstract

The extensive use of polypropylene (PP) in various industries has heightened interest in developing efficient methods for recycling and optimising its mixtures. This study focuses on formulating predictive models for the Melt Flow Rate (MFR) and shear viscosity of PP blends. The investigation involved characterising various grades, including virgin homopolymers, copolymers, and post-consumer recyclates, in accordance with ISO 1133 standards. The research examined both binary and ternary blends, utilising traditional mixing rules and symbolic regression to predict rheological properties. High accuracy was achieved with the Arrhenius and Cragoe models, attaining R^2^ values over 0.99. Symbolic regression further enhanced these models, offering significant improvements. To mitigate overfitting, empirical noise and variable swapping were introduced, increasing the models’ robustness and generalisability. The results demonstrated that the developed models could reliably predict MFR and shear viscosity, providing a valuable tool for improving the quality and consistency of PP mixtures. These advancements support the development of recycling technologies and sustainable practices in the polymer industry by optimising processing and enhancing the use of recycled materials.

## 1. Introduction

As of recent reports, approximately 19.7 million tonnes of plastic were used for packaging in Europe (EU27+3) in 2021, with PP constituting a substantial part with a share of roughly a quarter of this demand [1]. The growing use of plastics has escalated environmental concerns, propelling the importance of recycling to the forefront of global agendas. Effective recycling not only mitigates pollution but also conserves resources, aligning with the principles of a circular economy. In Europe, regulatory frameworks such as the EU Directive 2018/852 mandate that by the end of 2025, at least 50% of plastic packaging waste must be recycled, increasing to 55% by 2030 [2]. 

To achieve the targets of the EU directive, the use of recyclates must increase significantly over the next few years. It is necessary that recyclates can be flexibly adapted for use, even with strongly fluctuating properties and compositions, and they are in no way inferior to virgin material. However, recycling plastics, especially post-consumer recyclates, presents numerous challenges that are not solved yet. A significant issue is the contamination and variability in the composition of recyclates. Common contaminants in PP streams include materials like ethylene vinyl alcohol (EVOH), inks, and polyethylene (PE) [3]. These contaminants can adversely affect the properties of the recycled material, leading to fluctuations in viscosity and other critical parameters [3]. 

In general, a polymer’s viscosity is a measure of the resistance to flow and is crucial for processing and performance. It depends on a multitude of influences such as molecular weight, temperature, additives, and more [4,5]. The most important measurement of viscosity for injection moulding and extrusion processes is the shear viscosity, which describes the polymer’s resistance to flow when subjected to a parallel force. The shear viscosity can be measured using a capillary rheometer, where different shear rates can be set and the pressure difference in the capillary is measured and used for the viscosity calculation. For PP and most other polymers, the shear viscosity curve can be split into three distinct regions. At low shear rates, viscosity remains constant, indicating Newtonian behaviour. In the shear thinning region, the viscosity decreases with increasing shear rate due to polymer chains aligning in the flow direction. At very high shear rates, viscosity may level off or continue to decrease. Shear rates of 1 s^−1^ to 1000 s^−1^ are generally found in extrusion processes, while shear rates in processes such as injection moulding are in other orders of magnitude (100 s^−1^ to 100,000 s^−1^) [4]. Although the measurement of the shear viscosity curve provides detailed insights in the flow behaviour of polymers for processing conditions, the measurement requires costly equipment and is time consuming.

A simpler and more practical measurement widely used in the plastics industry is the MFR. It is measured by extruding molten polymer through a standardised die under a specific load and temperature and determining the mass of polymer extruded in a specified time frame of 10 min. Although the MFR gives only limited information and represents only a single point of the shear viscosity curve, the simplicity of this measurement is the reason for it being industry standard. 

Viscosity plays a determining role in polymer processing, influencing the efficiency and quality of manufacturing operations such as extrusion, injection moulding, and blow moulding [6,7,8,9]. Variability, especially in the shear viscosity of plastics, is particularly problematic. For instance, in injection moulding, an excessively low shear viscosity at the processing shear rates can lead to overfilling of moulds, while an excessively high shear viscosity might result in incomplete filling [6,7]. Both scenarios necessitate costly adjustments to the moulds or time-consuming alterations of the processing conditions. Similarly, film extrusion processes can only operate effectively within a specific viscosity range, making consistency essential to avoid defects and ensure smooth production [8]. 

In this field of research, many investigations are currently being carried out on the development of assistance systems or process adjustments to compensate for viscosity fluctuations in various processes. In injection moulding, for example, adapted process control strategies for the injection and holding pressure phases can ensure a constant process point even with fluctuating viscosities [10,11]. In the field of blown film extrusion, possibilities for compensating for viscosity fluctuations in the melt pre-distributor are being investigated [12]. Commercial solutions from system manufacturers are also available on the market [13]. However, the simplest solution for all manufacturing processes is the modification of the material, so that system retrofits would not even be necessary. This is particularly necessary for smaller plastics processors who cannot afford the investment costs for new assistance systems or plants usually required to achieve a smooth increase in recyclate utilisation rates.

## 2. Adjustment of Viscosity by Developing New Compound Recipes

To address the variability in viscosity, current strategies in compounding include blending different batches of recyclates or virgin polymers or adding specific additives to modify the viscosity [14,15,16]. These approaches help in achieving more consistent material properties, facilitating smoother processing and better-quality products.

### 2.1. Process of Recipe Development in the Industry

The development of a new formulation or the adjustment of an existing formulation rely mostly on compounders with years of experience and expertise. While invaluable, this empirical and iterative approach lacks reproducibility and is a meticulous and resource-intensive endeavour due to the necessity of compounding material samples, producing test specimens and performing the measurements to evaluate the success of the proposed formulation. Especially for minor adjustments of existing formulations, to compensate for varying input qualities and viscosities of recyclate streams, a more systematic, data-driven approach is of utmost importance [17,18,19]. Data-driven approaches utilising the capabilities of numerous machine learning methods such as Artificial Neural Networks (ANNs) have been proven to be promising in assisting the development of new compound recipes. Lopez-Garcia et al. compared different machine learning models for the development of fibre reinforced compounds incorporating recycled fibres and reached R^2^ values of up to 0.96 for the mechanical property prediction quality of the trained models [20]. Other investigations proved the effectiveness of ANNs to develop new compound formulations, achieving the specified colour values and impact strength of polyamides [21]. However, all of these data-driven approaches relied on datasets with a wide variation of the individual recipe components, fully documented process setups, and fully characterised compound properties. An application of such data-driven approaches in companies without such complete documentation is not as easy. Extensive trials would have to be performed to build an initial database, but the effort and cost to do so outweigh the benefit of reducing necessary development iterations.

Preliminary studies at the institute for plastics processing in Aachen, Germany showed how formulation parameters for an initial database of unknown formulation components can be identified as efficiently as possible when there is a lack of information density or no knowledge at all about the interaction of different polypropylenes and additives in a compound formulation [18]. Various Design of Experiments (DoE) strategies were investigated for two types of polypropylenes that were processed with a peroxide masterbatch in various mixing ratios at different processing temperatures and rotation speeds in a twin-screw extruder. With each investigated DoE, an ANN was trained to predict the MFR and mechanical properties of the compounds. After benchmarking each model on a separate validation dataset, it was proven that for this example the amount of necessary practical trials could be minimised by choosing a Definitive Screening Design for gathering initial data while still reaching R^2^ values of 0.97 for predicting the MFR [18]. 

### 2.2. Mixing Rules for Polymer Blends

While data-driven approaches prove to be effective for complex recipes with a multitude of components, the interaction of polymer blends can be modelled with simple mixing rules. To calculate the resulting MFR of binary blends, several investigations on a variety of mixing rules have been performed in the past. Some of these mixing rules have been intensively analysed for a multitude of virgin polymers [22,23] and several post-consumer recyclates [16]. Further investigations on modelling mechanical and rheological properties were conducted on a multitude of blends compounded with homopolymers, random copolymers, and bloc copolymers [24]. Similar investigations were performed for the shear viscosity [15,25,26,27,28]. Fisches et al. investigated several mixing rules for compositions of a polystyrene (PS) with polystyrene-based masterbatches and found simple linear mixing rules to perform the best [28]. Kneidinger et al. investigated shear viscosity mixing rules for binary blends with PP and blends with PP and polyamid (PA) with and without compatibilization. They confirmed the linear mixing rule to work properly for homogenous blends and showed that the linear mixing rule does not apply to the blend of PP and PA without compatibilization [25]. Dunkhin et al. investigated mixtures of Newtonian and non-Newtonian fluids at a fixed shear rate and found the linear mixing model with volume-based percentages to work surprisingly well for a combination of fluids with different properties [26]. 

In addition to the usage of various well-known physical models to describe the interactions between individual formulation components to predict properties of the compounds produced, symbolic regression is becoming increasingly popular [16,29,30,31,32,33]. Here, the relationships between the material compositions and the target variables are put together using a combination of different mathematical sub-models. In the past, such models have already been used in material development. Zhao et al. used symbolic regression models to determine the hardness and other mechanical properties of superhard materials [31]. Wang et al. demonstrated the potential of symbolic regression models for the purely data-based retrieval of known formulae to describe material behaviour [30]. Burlac et al. successfully used symbolic regression to model materials at the atomic level to avoid the high computational cost of simulation [29]. Symbolic regression has also already been used in plastics processing or mixing rules for plastics [16,32,33]. Pachner et al. predicted the pressure loss of non-Newtonian polymer melts through melt filtration systems, and Roland et al. were able to predict viscous dissipation of three-dimensional non-Newtonian flows in single-screw extruders [32,33]. Traxler et al. used it to improve the known models for predicting the MFR of binary blends for their data set [16].

### 2.3. Aim of the Research Presented in This Paper

Whether creating a new formulation to achieve specific rheological properties or adapting an existing one to accommodate varying input materials, such as different recycling batches, it is crucial to have a model that can predict the resulting rheological properties of the formulation. Although data sets are often available in a company, they are generally not originating from complex statistical design of experiments. A pragmatic approach to utilise the existing data is necessary to achieve the goal of formulating new recipe developments or adapting existing ones due to fluctuating input material qualities. 

Within this paper, both the prediction of the MFR and shear viscosity with simple mathematical models is investigated. For this purpose, binary blends consisting of two different types of PP are considered first. As not all data is necessarily always available in practical applications, the established prediction models for the MFR and shear viscosity are extended using a pragmatic best-fit approach so that they can be used on sparse data sets. After confirming the validity of this approach for binary blends, the models are extended to be applicable for arbitrarily complex blends. The practical testing of these extended models is conducted with ternary blends (blends with three components). Finally, a pragmatic modelling approach is made available that is primarily designed for use in industrial companies.

## 3. Experimental

To gain experimental data regarding the compound properties in dependence of arbitrary blend mixtures and polymer types to predict the resulting MFR and shear viscosity curves, various polymers are identified.

### 3.1. Materials and Characterisation

For the experiments, three virgin homopolymer PP grades, two virgin copolymer PP grades, and one commercially available PP post-consumer recyclate (PCR) were used. The materials were provided by Borealis (Vienna, Austria), Lyondell Basell (Rotterdam, The Netherlands), Saudi Basic Industries Corporation (SABIC) (Riyadh, Saudi Arabia), and Systec Plastics GmbH (Cologne, Germany). The MFR values from the data sheets were measured according to ISO 1133 with parameters typically used for PP: a temperature of 230 °C and a weight of 2.16 kg [34]. The shear viscosity was measured at a temperature of 230 °C with three round capillaries (diameter of 1 mm) with lengths of 20 mm, 10 mm, and 5 mm on a high-pressure capillary rheometer of the type RHEOGRAPH 50 by GÖTTFERT Werkstoff-Prüfmaschinen GmbH (Buchen, Germany). The three different capillaries were used to perform the Bagley correction to compensate for inlet and outlet pressure losses [35]. 

In the subsequent investigations, materials are designated by abbreviations reflecting: (i) their source, with ‘v’ indicating virgin and ‘c’ for PCR; (ii) the type of virgin polypropylene, where ‘H’ denotes homopolymer, ‘B’ denotes block copolymer, and ‘R’ represents a recyclate mix; and (iii) their MFR value as measured. Table 1 presents a summary of the materials evaluated in this research.

### 3.2. Laboratory Equipment for Compounding

The compounding of all materials was performed on a co-rotating twin-screw extruder (Coperion GmbH, Stuttgart, Germany) with a screw diameter of 26 mm and a targeted melt temperature of 210 °C. The composition of the screw elements consisted of only conveying screw elements with a combination of kneading and mixing elements used in the beginning of the process to plasticise the polymers. The same process setup was used for all blending trials. The compounder rotational speed was kept at 300 min^−1^ and the material throughput was fixed at 15 kg/h. The composition of the binary blends and the designated blend names can be seen in Table 2. For each of the six blends, a total of 10 compositions was produced. The chosen blend compositions can be seen in Table 3. The test points were not selected with equidistant steps of the component shares. In the experiments by Traxler et al. no inconsistencies were found in the model behaviour for large component proportions [16]. To check the models for smaller mixture proportions, the focus in these experiments was therefore placed on more sampling points for small mixture proportions. For the compounding of ternary blends, the blend compositions can be seen in Table 4.

## 4. Application of Mixing Rules for the Prediction of the MFR

A model-based prediction for the MFR of binary blends is possible by applying simple mixing rules. In the following, the Arrhenius and Cragoe mixing rules will be investigated for the binary blends. Afterwards, a fitting approach to match sparse datasets with these mixing rules will be described before more complex symbolic regression models are applied on the data set. Lastly, the application of the mixing rules for ternary blends is investigated.

### 4.1. Modelling the MFR of Binary Blends with Traditional Mixing Rules

**Figure 1:**
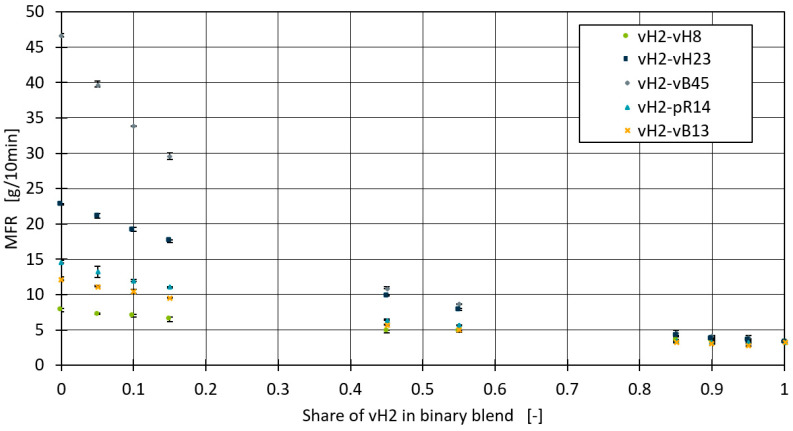
MFR measurements of all blends in Set 1.

For the measurement of the MFR, three measurements were carried out for each of the test points. The average measurement error was 0.168. Figure 1 shows the measured MFR values of the binary blends. For all of the blends, there is a clear trend where the MFR generally decreases with an increased proportion of vH2 in the blend, as would be expected since an increase of the blend partner with lower MFR should result in lower overall mixture MFR values. When the vH2 content reaches 0.1 and below, the MFR shows a significant decrease, especially in the vH2–vB45 and vH2–vH8 blends. 

Following an intensive review of different mixing rules for PP blends by Traxler et al., the Arrhenius and Cragoe mixing rules were selected as initial models for the blends produced in this investigation due to their high predictive power [16]. The Arrhenius model (Equation (1)) describes the logarithmic MFR of a mixture to be the sum of the logarithm applied on each individual blend partner’s MFR multiplied by its share in the blend [36]. The Cragoe model (Equation (2)), on the other hand, states that the reciprocal of the logarithm of the MFR is the sum of the individual reciprocals of the MFRs of the blend partners multiplied by their proportion in the blend [37].

(1) $$\ln\left({MFR}_{mix}\right)=x_{1}\ln\left({MFR}_{1}\right)+x_{2}ln({MFR}_{2})$$

(2) $$\frac{1}{ln(L\;{MFR}_{mix})}=\frac{x_{1}}{ln(L\;{MFR}_{1})}+\frac{x_{2}}{ln(L\;{MFR}_{2})}$$

**Figure 2:**
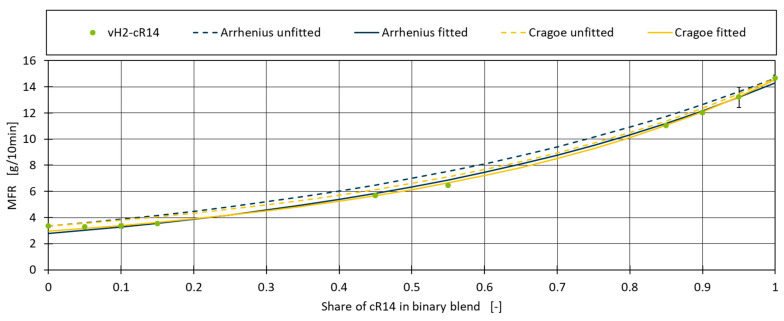
Application of fitted and unfitted mixing rules on blend vH2–cR14.

The constant L in the Cragoe mixing rule is mainly dependent on the type of liquid to which the mixing rule is applied on and was set to 2000 as suggested by Gao and Li [38]. Figure 2 shows the application of both models to the vH2–cR14 blend. Although both models roughly match the shape of the measurements, the predicted MFR values for the different blends are consistently overestimated.

A common problem with all mixing rules is that only the MFR values of the base materials are used to calculate the mixtures. Therefore, small deviations in the measurements of the raw materials can lead to large deviations in the different compound calculations. Furthermore, if such mixture models are to be applied to existing data in a company producing different mixtures, the raw material data may not necessarily be available. Therefore, we propose a best fit approach for determining the parameters ${MFR}_{1}$ and ${MFR}_{2}$ according to Equation (3). Here, $f_{{MFR}_{mix,\;i}}$ may be any viscosity model capable of calculating the MFR of a binary mixture and n is the number of samples provided.

(3) $$\underset{{\mathrm{MFR}}_{1},{\mathrm{MFR}}_{2}}{\min}\sum_{i=1}^{n}{\left[{MFR}_{mix,\;i}-f_{{MFR}_{mix,\;i}}(x_{1,i},x_{2,i},{MFR}_{1},{MFR}_{2})\right]}^{2}$$

It must be noted that in this case the quadratic deviation between prediction and ground truth was chosen to optimise the parameters. Depending on the application scenario, different criteria can be chosen (e.g., minimising the percentage deviation between prediction and ground truth).

In applying this calculation to the Arrhenius and Cragoe models for blend vH2–R14, the fitted values for ${MFR}_{1}$ (cR14) was calculated to be 14.32 for Arrhenius and 14.54 for Cragoe model compared to the measured value of 14.67. For ${MFR}_{2}$ (vH2), the values are 2.80 and 2.97 compared to the measured value of 3.34. The blend percentages used within the model remain unchanged. Using the fitted values, it can be seen in Figure 2 that now both the Arrhenius and Cragoe models match almost perfectly with the real data. To quantify the improvement of this approach and to measure the performance of the models, the Mean Absolute Error (MAE) and the Coefficient of Determination (R^2^) are calculated.

(4) $$MAE=\frac{1}{n}\sum_{i=1}^{n}\left|{MFR}_{prediction,\;i}-{MFR}_{measured,\;i}\right|$$

(5) $$R^{2}=1-\frac{{\left({MFR}_{prediction,\;i}-{MFR}_{measured,\;i}\right)}^{2}}{{\left({MFR}_{mean,\;i}-{MFR}_{measured,\;i}\right)}^{2}}$$

The *R*^2^ value is a statistical measure that represents the proportion of the variance for a dependent variable that is explained by an independent variable or variables in a regression model. An R^2^ of 1 indicates that the regression predictions perfectly fit the data. Conversely, an R^2^ of 0 indicates that the model does not explain any of the variance. A low R^2^ value does not necessarily mean the model is inadequate; it could indicate a high level of inherent variability in the data or that the model is applied in a field with high variability. However, a comparison of the R^2^ value for models applied on the same data set provides valuable information on the model capabilities. To quantify the prediction quality with regards to prediction error, the MAE is calculated.

By applying the fit of ${MFR}_{1}$ and ${MFR}_{2}$ on the different blends, the MAE can be decreased and the R^2^ can be increased. All calculated errors and model scores can be seen in Table 5. For all the different blends, an R^2^ greater than 0.992 was obtained. In terms of model performance, the Cragoe model performed best for all blends except vH2–vB45.

### 4.2. Modelling the MFR of All Blends Utilising Symbolic Regression

Even though the MAEs for the fitted Cragoe model are rather small, with 0.370 being the maximum prediction error for the blend vH2–vB45, a further improvement of the models is necessary. When modelling the viscosity of more complex blends consisting of a multitude of components, the traditional binary models must be applied stepwise. For an exemplary blend of four polymers, the binary models need to be applied three times. According to Gaussian error propagation, the combined error (σ_y_) of a model prediction can be calculated by applying Equation (6) [39] as follows:

(6) $$\sigma_{y}=\sqrt{\sum {\left(\frac{\partial y}{\partial x_{i}}\right)\sigma_{x_{i}}}^{2}}$$

In this equation $x_{i}$ represents the individual input variables and $\frac{\partial y}{\partial x_{i}}$ is the partial derivative of the output y with respect to the input variable. For the assumption that the error of the binary model is always the same, applying it for the model with the lowest MAE (0.120 for Blend vH2–vH8) would lead to an increased MAE of 0.170. For the highest prediction error that was found (0.370 for Blend vH2–vB45), the MAE would increase to 0.641 for a composition of four polymers. Furthermore, when additives or fillers with individual models for each are applied, the error would increase only more. Therefore, even though the prediction accuracy is relatively high, a further increase in prediction accuracy is necessary.

**Figure 3:**
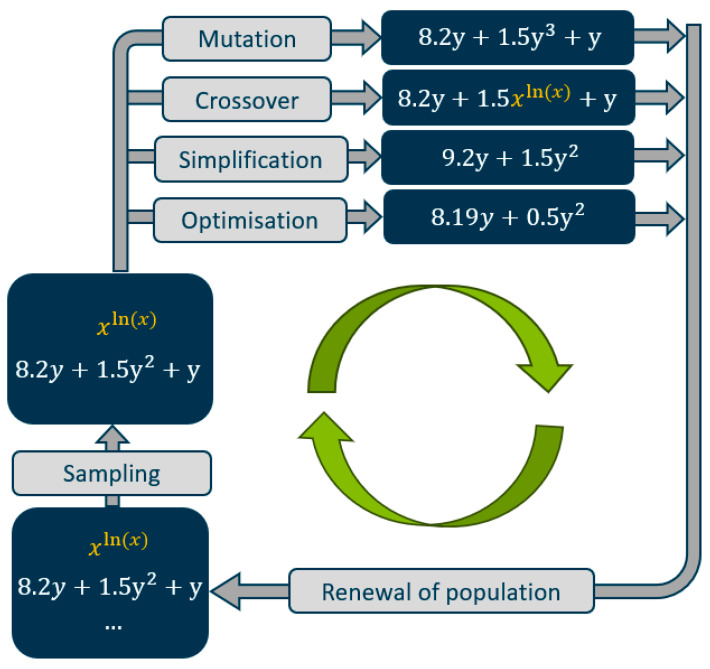
Working principle of symbolic regression using PySR.

Symbolic regression (SR) is a type of machine learning that aims to discover human-interpretable symbolic models from data. Unlike traditional regression techniques, which fit parameters within a predetermined model structure, SR explores a large space of possible mathematical expressions to identify the best-fitting model [29,32,33,40]. The Python based PySR framework, which uses the SymbolicRegression.jl backend, facilitates this process through a multi-population evolutionary algorithm [40]. This algorithm involves several key steps, as illustrated in Figure 3.

First, a population of random mathematical expressions is chosen. From this population, the fittest individuals are selected based on a fitness function. By applying genetic operators such as mutation, crossover, and simplification, the individuals evolve towards a population that provides better solutions. The evolutionary loop is further enhanced by simulated annealing, age-regulated evolution, and a unique evolve–simplify–optimise cycle that iteratively refines both the structure and constants of the expressions [40,41].

For modelling the binary blends with and without additives, it was found that the Arrhenius and Cragoe models already achieve high model scores (R^2^). Building on this finding, the PySR framework can be used to derive improved mixing rules for polymer blends by focusing on sub-components of this formula, such as $x_{1}\ln\left(x_{2}\right)$, alongside other fundamental operations like linear, exponential, and logarithmic functions. To reduce the risk of overfitting, which occurs when a model learns the noise and random fluctuations in the training data rather than the underlying pattern, the dataset was expanded fivefold by introducing an empirically chosen gaussian noise of 0.1% and by swapping the input variables ($x_{1}$, $x_{2}$, ${MFR}_{1}$, ${MFR}_{2}$) to ensure the robustness and bidirectional applicability of the derived mixing rules. This approach helps ensure that the final symbolic models will generalise well and reflect the underlying physical principles of polymer blend behaviour. After training with the binary blend data and choosing the equation with the minimum complexity setting of PySR (least number of mathematical expressions in the formula), Equation (7) was found.

(7) $${MFR}_{SR-Model}=e^{x_{1}*\log\left({MFR}_{1}-1.213\right)+x_{2}*\log\left({MFR}_{2}-1.18\right)}+1.25$$

**Figure 4:**
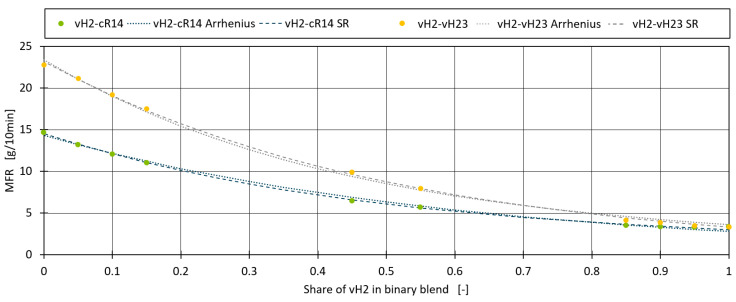
Symbolic regression model applied to the binary blends vH2–cR14 and vH2–vB23.

The symbolic regression model for calculating the MFR of a binary mixture is very similar to the Arrhenius model, except for the additional fitting coefficients that adapt the model to the given data set (−1.213, −1.18, and +1.25). Compared to the symbolic regression model found by Traxler et al., the model in Equation (7) is much simpler and applicable in both blending directions (blending the higher MFR component with the lower MFR component as well as vice versa) [16]. The model is applied to the two-blend datasets vH2–cR14 and vH2–vH23 in Figure 4. The symbolic regression model fits the data better than the fitted Arrhenius model with an MAE of 0.141 instead of 0.187 for vH2–cR14 and 0.172 instead of 0.214 for vH2–vH23.

To evaluate the overall performance of the newly found model and to compare it with both the Arrhenius and Cragoe models, the MAE of the applied models were calculated on all blends. The parameters of ${MFR}_{1}$ and ${MFR}_{2}$ were determined for all models according to the optimisation approach described in Section 4.1. In addition, to compare the approach presented in this paper to previous research, a further evaluation was carried out on the full dataset used by Traxler et al. [16]. The results of this evaluation are shown in Table 6.

For the binary blends of this investigation, the symbolic regression model gives the lowest MAE of 0.217 compared to the Arrhenius model (0.280) or the Cragoe model (0.233). The symbolic regression model may fit the dataset on which it was trained better than the standard Arrhenius and Cragoe models. This can also be seen when all models are applied to the dataset studied by Traxler et al. Here, the symbolic regression model (MAE of 0.266) is worse than Arrhenius (0.195) and Cragoe (0.165) [16]. Nevertheless, when the model is compared with the error in the measurement of the MFR itself (0.168) for the binary blend dataset it was trained on, there is not much room for further improvement. A difference of 0.1 in the MFR value between prediction and measurement should be sufficient for most applications.

### 4.3. Application of the Mixing Rules on Ternary Blends

Compounds for industrial applications contain a variety of different materials. In addition to pure virgin compounds, different additives may be present in the form of masterbatches, consisting of minimally chemically active ingredients mixed in a polymer to make dosing in the final compounds easier. Therefore, the adaption of the mixing rules on blends with a multitude of blend partners is important. One possibility to apply mixing rules such as those in the Arrhenius model to these additive blends is the stepwise application discussed in Section 4.2. Another possibility, eliminating the individual model errors adding up for each application, is to extend the Arrhenius model to be applicable for blends with arbitrary components in one calculation. To apply such a generalised model on the data of Table 4, the Arrhenius model for binary blends was extended to the generalised Equation (8) with $x_{i}$ and ${MFR}_{i}$ being the shares and MFR values of the individual blend partners, with the requirement that the sum of all $x_{i}$ equals one.

(8) $$\ln\left({MFR}_{mix}\right)=\sum_{i}^{3}x_{i}\ln\left({MFR}_{i}\right)$$

Applying Equation (8) directly to the data of the ternary blends, an R^2^ of 0.991 and an MAE of 0.923 was obtained. With more than two blending partners, the problem of imprecise measurements of the pure materials’ MFR values or the problem of missing measurements in data available in a company as described in Section 4.1 remains and may be greater due to the addition of individual errors. Therefore, Equation (3) can be adapted to the generalised Arrhenius formula as follows, where k is the number of blend partners:

(9) $$\underset{{\mathrm{MFR}}_{1},{\mathrm{MFR}}_{2},\ldots ,{\mathrm{MFR}}_{k}}{\min}\sum_{i=1}^{n}{\left[{MFR}_{mix,\;i}-f_{{MFR}_{mix,\;i}}(x_{1,i},x_{2,i},\ldots ,x_{k,i}{,MFR}_{1},{MFR}_{2},\ldots ,{MFR}_{k})\right]}^{2}$$

**Figure 5:**
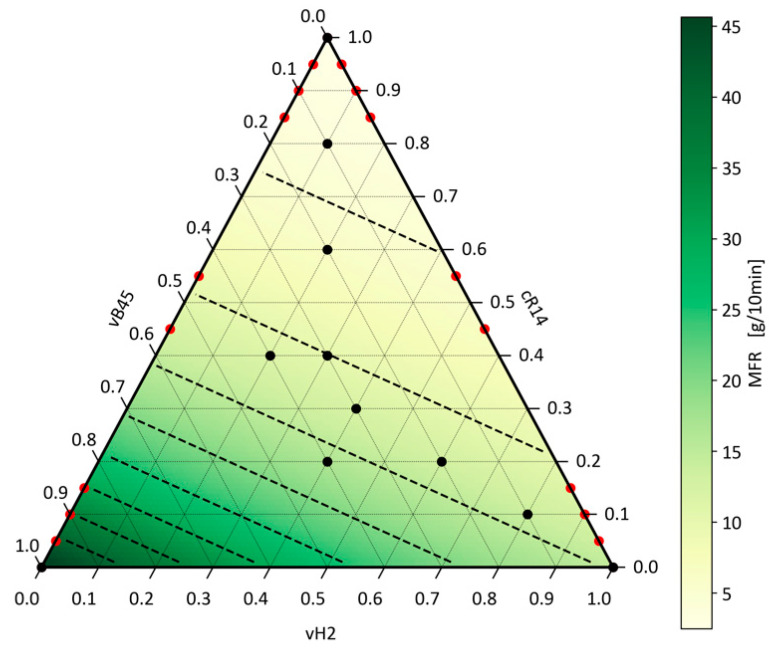
Application of the generalised Arrhenius mixing rule on ternary blends.

By applying Equation (9), the R^2^ of the unfitted Arrhenius model was increased to 0.999 instead of 0.991 and the MAE was reduced to 0.355 instead of 0.923. Since the blend partners used in this investigation ranged from MFR values of 2 to 45, and the polymers represented block and homopolymers as well as a commonly used recyclate, the applicability of the Arrhenius model to multiple-partner blends can be concluded for PP. The model prediction of Equation (8) with the optimised MFR parameters can be seen in Figure 5. The black dots indicate the experiments performed and measurements taken for the optimisation. The dashed black lines mark isolines with the same MFR values in steps of five. The logarithmic relationship of the MFR values can be seen by the distance between the isolines decreasing as the MFR increases. The data set used to fit the model for the ternary blends covers primarily test points in the centre of the parameter space. Therefore, the applicability of the model to the edges of the parameter space must also be checked. For this purpose, the fitted model is applied to the data series vH2–vB45 and vH2–cR14 from the previous investigations for binary blends. These points are marked in red in Figure 5. Despite fitting to the ternary data set, the MAE for these data points is 0.68 and the R^2^ value is 0.996.

### 4.4. Results of the Application of Mixing Rules for Predicting the MFR

The experimental data showed that the traditional Arrhenius and Cragoe models achieved high predictive accuracy without any adjustment, with R^2^ values exceeding 0.99 for some binary blends. By applying the proposed fitting method, the Arrhenius and Cragoe model were significantly improved. For the Arrhenius model, the prediction MAE was reduced to 0.280 from 0.467, and for the Cragoe model, a reduction in MAE to 0.233 from 0.309 occurred.

Furthermore, the application of the PySR framework allowed for further improvements. The symbolic regression model developed in this study yielded an R^2^ value of 0.999 and an MAE of 0.217, demonstrating superior performance compared to traditional models.

For ternary blends, the Arrhenius model was extended to accommodate multiple blend partners. The generalised Arrhenius model achieved an R^2^ value of 0.999 and an MAE of 0.36, indicating high predictive accuracy and supporting the hypothesis that these models can be applied to more complex blends. This extension to ternary blends suggests the robustness and applicability of these models for predicting MFR in blends with more than two components.

## 5. Application of Mixing Rules for the Prediction of the Shear Viscosity Curve

The interpretation of the MFR is rather limited, especially when it comes to the processability of a material in processes such as injection moulding or extrusion, this value is widely used in the industry due to the simple measurement method. Compared to the MFR, shear viscosity curves are more reliable. Similarly to the investigations performed in Section 4, known mixing rules for the shear viscosity of blends were tested. After this, the symbolic regression approach and the applicability on ternary blends was evaluated.

### 5.1. Modelling the Shear Viscosity Curve of Binary Blends

Measuring the shear rate curve using a high-pressure capillary rheometer is much more suitable for a more detailed interpretation of the flowability of a plastic compound during processing. For example, predicting the shear viscosity of a compound for a known processing shear rate in injection moulding can indicate whether the processing of said compound may result in viscosity related difficulties. The development of mixing rules for the shear viscosity analogue to the MFR value is therefore important. Investigations into different mixing rules for this case have been carried out for different polymer types or blends where PP is a blend partner. A mixing rule that is generally found to be the most suitable for this case is the linear mixing rule according to Equation (10) [26,28].

(10) $$\eta_{mix}\left(\dot{\gamma }\right)=x_{1}\eta_{1}\left(\dot{\gamma }\right)+x_{2}\eta_{2}\left(\dot{\gamma }\right)$$

The shear viscosity of the various blends of Table 2 and Table 4 was measured with fixed shear rates of 51 s^−1^, 102 s^−1^, 204 s^−1^, 408 s^−1^, 815 s^−1^, and 1630 s^−1^. However, for the investigation of the viscosity for specific processing shear rates an interpolation between the measurements may be necessary. To describe the shear viscosity curve, a multitude of models can be chosen. The most common models of Ostwald and DeWaele (Equation (11)) and Carreau (Equation (12)) were evaluated in our research to find the best fit [4].

(11) $$\eta \left(\dot{\gamma }\right)=K{\dot{\gamma }}^{n-1}$$

(12) $$\eta \left(\dot{\gamma }\right)=\frac{A}{{\left(1+B\dot{\gamma }\right)}^{C}}$$

**Figure 6:**
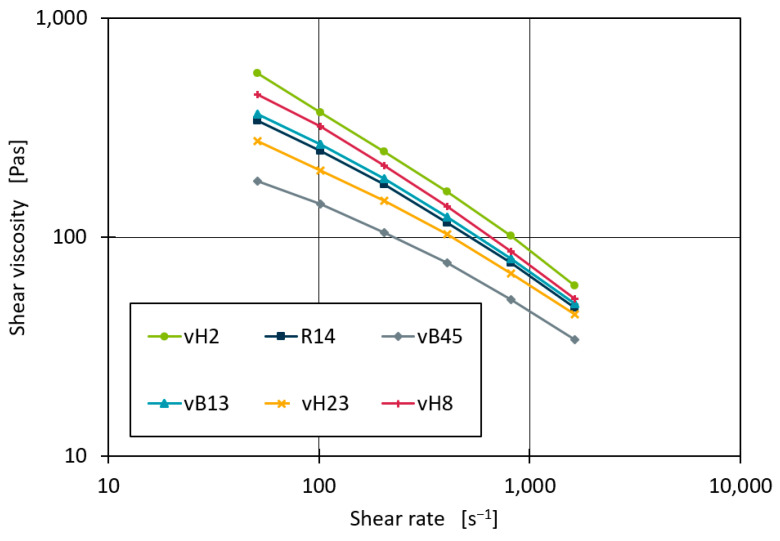
Measured shear viscosity curves of the base materials.

Similarly to Equation (3), the best parameters of these models to fit the measured data points of each compound were identified. With the optimised Ostwald–DaWaele parameters, average R^2^ scores of 0.9977 were obtained. However, the Carreau model achieved an average R^2^ of 0.9999. Figure 6 shows the measured shear viscosity curves for the pure base materials of dataset 1 on a double logarithmic scale. The shape of the curves, especially for the lower shear rates, is not strictly linear but slightly curved. This may indicate that within the measurements the transition area of Newtonian fluid behaviour to structural viscosity behaviour was measured. The Ostwald–DaWaele model is best suited to describing only the behaviour of fluids with structural viscosity. However, the Carreau model can capture the transition point as well as the Newtonian regions [4]. This explains the better performance of the Carreau model for the data fit. Therefore, the following investigations are based on the Carreau model. However, it should be noted that the fitted Carreau parameters are only valid for shear rates of 51 s^−1^ up to 1630 s^−1^.

**Figure 7:**
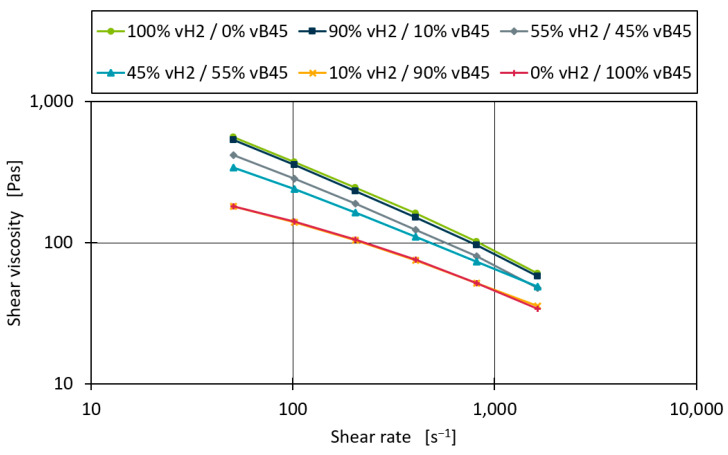
Shear viscosity curves for the blend series vH2–vB45.

Figure 7 shows the shear viscosity curves for the measurements of the binary blend vH2–vB45. The blends with 10% and 90% of both blending partners do not deviate much from the pure material measurements. For the 45% and 55% blends the measurements are close to the centre between the base material curves throughout the complete shear rate range.

Similar to the method used for the MFR, the shear viscosity curve of the base material was optimised to minimise the quadratic loss between the model viscosity prediction and the measurements according to Equation (13).

(13) $$\underset{A_{1},B_{1},C_{1},A_{2},B_{2},C_{2}}{\min}\sum_{\dot{\gamma }=50}^{1630}\sum_{i=1}^{n}{\left[\eta_{mix,i}\left(\dot{\gamma }\right)-f_{\eta_{mix,i}}(x_{1,i},x_{2,i},A_{1},B_{1},C_{1},A_{2},B_{2},C_{2},\dot{\gamma })\right]}^{2}$$

**Figure 8:**
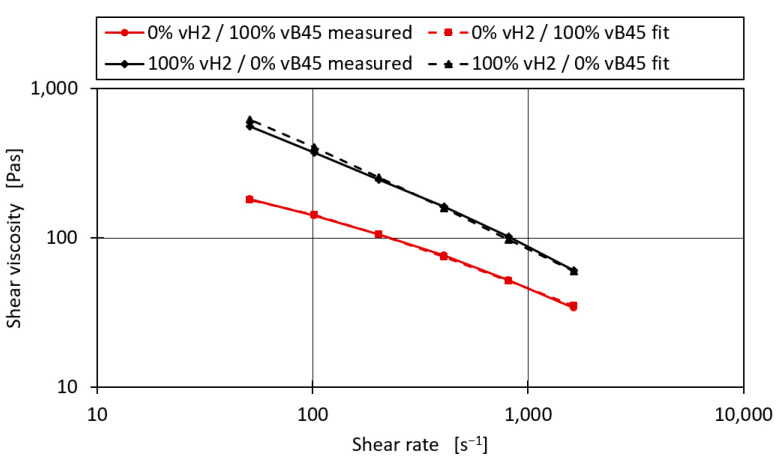
Optimised shear viscosity curves for the blend vH2–vB45.

An example of the optimised Carreau parameters and the resulting shear viscosity curve can be seen in Figure 8 for the blend series vH2–VB45. After applying the optimisation to the entire mixture series and calculating the MAE and R^2^ of both the fitted and unfitted linear models, it can be seen that an R^2^ of 0.989 was obtained for the binary mixtures compared to the R^2^ of 0.977 without applying the fit. Similarly, the MAE was reduced to 10 from the 12.31 without applying the fit. The R^2^ values do not reach the R^2^ values of 0.99 and above as it did for the MFR. This may be due to the fact that the shear viscosity prediction model is more complex, with the shear rate itself being a critical parameter, as well as the three parameters A, B, and C needed to describe the base material. However, an average prediction error of 10 Pas for the measured shear viscosities ranging from 34.1 Pas to 654.7 Pas may be sufficient for industrial application.

### 5.2. Modelling the Shear Viscosity Curve with a Symbolic Regression Model

To investigate the maximum model quality achievable with the data gathered, similarly to Section 4.2, a symbolic regression model was trained. The data augmentation process with a fivefold increase of the dataset and the application of an empirically chosen Gaussian noise of 0.1% was kept the same. Furthermore, the swapping of the input variables to ensure the bidirectional applicability like the linear model was conducted. The model found after this application is given in Equation (14).

(14) $$\eta_{SR-Model\;}\left(\dot{\gamma }\right)={1.0929(x}_{1}\eta_{1}\left(\dot{\gamma }\right)+x_{2}\eta_{2}\left(\dot{\gamma }\right))-7.8885$$

Very similar to the symbolic regression model for the MFR in Section 4.2, the same equation as the base model was found with minor correction coefficients to fit the original dataset. By applying this model, the MAE was reduced to 7.91 and the R^2^ value was increased to 0.993. As the root mean square error was minimised in the optimisation process, the symbolic regression model fits better at lower shear rates compared to the linear model.

### 5.3. Modelling the Shear Viscosity Curve of Ternary Blends

**Figure 9:**
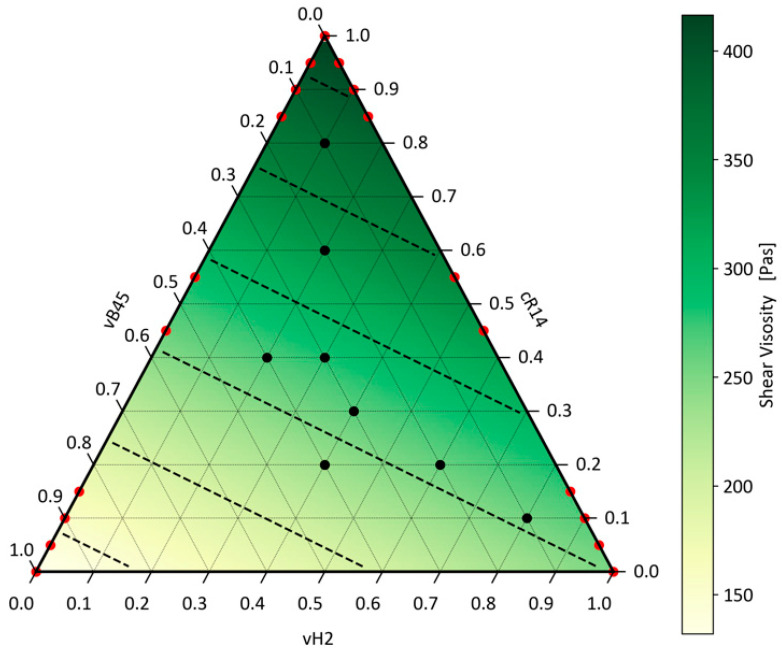
Application of the generalised linear mixing rule on ternary blends with a shear rate of 100 s^−1^.

To assess the applicability of the linear mixing model to blends with any number of mixing partners, the model was also evaluated on the ternary blends. Therefore, Equations (10) and (13) were extended similarly to the procedure for the MFR value. For the ternary blend, an MAE of 1.99 and an R^2^ of 0.993 were obtained, indicating that the linear model can be applied to any PP blend. The visualisation of the model predictions for a given shear rate of 100 s^−1^ is shown in Figure 9.

Similarly to the application of the model on the MFR data, the fit was conducted on test points mostly located within the centre of the parameter space. Therefore, the model was also evaluated on the vH2–vB45 and vH2–cR14 data series from the previous investigations for binary blends. Here, an R^2^ of 0.98 was achieved.

Analogous to the visualisation of the Arrhenius rule for the ternary mixtures with the prediction of the MFR value, the isolines represent the mixtures with constant shear viscosity in steps of 50 Pas. The distance between the isolines is always the same compared to the logarithmically decreasing distance as for the MFR model, showcasing the linear interaction.

## 6. Discussion

The application of mixing rules to adjust the MFR and shear viscosity of PP compounds is crucial for enhancing the quality and consistency of recycled materials. Conventional mixing rules, such as the Arrhenius and Cragoe models for the MFR and the linear model for shear viscosity, have demonstrated their utility in blending virgin polymers in binary blends. However, the application of these models to blends involving more than two components has not been thoroughly explored.

Regardless of the mixing model applied to predict the MFR or shear viscosity of a blend, it is essential to have the MFR values or shear viscosity data of the pure materials used in the blend. Consequently, these models cannot be directly applied to datasets containing only blended components without test points of the pure materials.

To address this limitation, we proposed an approach to fit these parameters to existing data. By selecting the characteristic values of the starting materials to minimise the error between the model prediction and the actual blend values, it becomes possible to apply these models to all datasets. Even when the characteristic values of the pure materials are available, our study demonstrated that the prediction error for blend series can be further minimised using this best-fit approach. Consequently, the prediction error (MAE) of the classical Arrhenius and Cragoe models for MFR, as well as the prediction error of the linear mixing rule for shear viscosity curves, was significantly reduced for binary blends through this fitting method.

Symbolic regression allows for further adaptation of existing models to the available dataset, thereby improving prediction accuracy. However, achieving a general improvement in prediction accuracy across a diverse range of datasets with varying characteristics is not feasible. Specifically, for the symbolic regression model for MFR, simultaneous optimisation for datasets with and without additives was not possible. A comparison with reference datasets also revealed that classical models with fitted material parameters are superior when applied on arbitrary datasets.

In addition to applying the Arrhenius model and the linear mixing rule for shear viscosity to binary blends, we investigated the extension of these models to ternary blends. Our findings indicate that these models can be applied without restrictions and with similar prediction accuracies as for binary blends, suggesting their potential applicability to blends with more than three components.

The individual MAE and R^2^ values can be compared in Table 7.

## 7. Conclusions

This study thoroughly examined the applicability of mixing rules for predicting the Melt Flow Rate and shear viscosity of polypropylene compounds. Employing a comprehensive experimental framework, we assessed binary and ternary blends of virgin and post-consumer polypropylene. Traditional mixing rules, specifically the Arrhenius and Cragoe models for Melt Flow Rate and a linear model for shear viscosity, were evaluated and optimised to reduce prediction errors.

Our findings indicated that while classical models provided substantial predictive power, their accuracy significantly improved when the parameters of the base materials were fitted to minimise the error between model predictions and actual measurements. Furthermore, symbolic regression was employed to develop more refined models, further enhancing prediction accuracy for specific datasets.

For ternary blends, the Arrhenius model for Melt Flow Rate and the linear model for shear viscosity demonstrated high predictive accuracy, suggesting their robustness and applicability to blends with more than two components.

Future research will aim to validate the applicability of these mixing rules on a broader range of polypropylene blends, including different copolymer types and blends involving other polymers. Other additives typically used in PP-compounds such as chalk, talcum, or impact strength modifiers will be investigated as well.

Additionally, further refinement of the symbolic regression models will be pursued to enhance their general applicability and predictive accuracy. This ongoing research is crucial for advancing recycling technologies and supporting sustainable practices within the polymer industry by ensuring the quality and consistency of recycled polypropylene materials.

## Figures and Tables

**Table 1:** Materials used in the investigations.

Designation	Material	MFR
vH2	505P	2.0
vH8	HP501M	7.5
vB13	BE170CF	13.0
vH23	HP548R	23.0
vB45	FPC45	45.0
cR14	Systalen PP–C24000 gr000	14.0

**Table 2:** Compositions of binary blends used in the investigations.

Blend Name	Blend Partner 1					Blend Partner 2
	vH8	vB13	vH23	vB45	pR14	vH2
vH2–vH8	X	-	-	-	-	X
vH2–vB13	-	X	-	-	-	X
vH2–vH23	-	-	X	-	-	X
vH2–vB45	-	-	-	X	-	X
vH2–cR14	-	-	-	-	X	X

**Table 3:** Blend composition of all binary blends.

X_1_	0%	5%	10%	15%	45%	55%	85%	90%	95%	100%
X_2_	100%	95%	90%	85%	55%	45%	15%	10%	5%	0%

**Table 4:** Blend composition for the second set of trials for the ternary blends.

	2.1	2.2	2.3	2.4	2.5	2.6	2.7	2.8	2.9	2.10	2.11
vH2	100%	80%	60%	40%	20%	0%	10%	20%	30%	40%	0%
cR14	0%	10%	20%	30%	40%	100%	80%	60%	40%	20%	0%
vB45	0%	10%	20%	30%	40%	0%	10%	20%	30%	40%	100%

**Table 5:** MAE and R^2^ of the Arrhenius and Cragoe model for the binary blends.

Blend	Measurement	Arrhenius Unfitted		Arrhenius Fitted		Cragoe Unfitted		Cragoe Fitted	
	Std. Dev.	MAE	R^2^	MAE	R^2^	MAE	R^2^	MAE	R^2^
vH2–vH8	0.173	0.166	0.980	0.136	0.990	0.134	0.987	0.120	0.992
vH2–vH23	0.142	0.243	0.998	0.187	0.999	0.556	0.990	0.431	0.996
vH2–vB45	0.202	1.124	0.990	0.675	0.998	0.372	0.999	0.370	0.999
vH2–R14	0.176	0.514	0.980	0.214	0.996	0.335	0.992	0.214	0.996
vH2–vB13	0.149	0.286	0.991	0.187	0.994	0.150	0.997	0.131	0.997

**Table 6:** MAE and measurement deviation for all different models and datasets.

	Average Deviation	Average MAE		
Data Source	Measurement	Arrhenius Fitted	Cragoe Fitted	Symbolic Regression
Our data	0.168	0.280	0.233	0.217
Traxler et al. [16]	0.094	0.195	0.165	0.266

**Table 7:** MAE and R^2^ values of the fitted Arrhenius and symbolic regression model for the various datasets investigated within this paper.

	MFR					Shear Viscosity			
Dataset	Measurement Deviation	Arrhenius Mixing Rule		Symbolic Regression		Linear Mixing Rule		Symbolic Regression	
		MAE	R^2^	MAE	R^2^	MAE	R^2^	MAE	R^2^
Binary blends	0.168	0.28	0.999	0.23	0.999	10.0	0.989	7.91	0.993
Ternary blends	0.158	0.36	0.999	x	x	2.0	0.993	x	x
Traxler et al. [16]	0.094	0.20	0.998	0.27	0.996	x	x	x	x

## Data Availability

The original contributions presented in the study are included in the article, further inquiries can be directed to the corresponding author/s.
